# Suppression of ^18^F-FDG signal in the bladder on small animal PET-CT

**DOI:** 10.1371/journal.pone.0205610

**Published:** 2018-10-17

**Authors:** Lorena Cussó, Manuel Desco

**Affiliations:** 1 Departamento de Bioingeniería e Ingeniería Aeroespacial, Universidad Carlos III de Madrid, Madrid, Spain; 2 Instituto de Investigación Sanitaria Gregorio Marañón, Madrid, Spain; 3 Centro de Investigación Biomédica en Red de Salud Mental (CIBERSAM), Madrid, Spain; 4 Centro Nacional de Investigaciones Cardiovasculares Carlos III (CNIC), Madrid, Spain; Biomedical Research Foundation, UNITED STATES

## Abstract

**Introduction:**

Retention of 2-deoxy-2-[^18^F]fluoro-D-glucose ^18^F-FDG in the bladder causes more problems in small animal research than in human research owing to the smaller size of the subject. Catheterization has been proposed to reduce bladder spillover both in human studies and in small animal research. Noninvasive alternatives such as hydration plus furosemide also seem to be a promising pre-imaging strategy for decreasing bladder spillover. Our main goal was to measure the effects of the combination of furosemide and hydration for reducing bladder signal directly on mouse bowel ^18^F-FDG-PET images.

**Methods:**

Nine mice were divided into two groups: the control group (C, n = 4) and the treatment group (n = 5). The clearance protocol combines hyperhydration and a single furosemide dose during the ^18^F-FDG uptake period. Two images were acquired on different days in treated mice to evaluate two different furosemide doses (low dose, LD, 3.5 mg/kg; and high dose, HD, 7 mg/kg). A region of interest was drawn on each computed tomography image (bladder, kidneys, liver, muscle, and bone marrow). To quantify bladder spillover, two different areas of the colon were selected.

**Results:**

A remarkable reduction in bladder spillover was achieved on ^18^F-FDG -PET in both groups. Our imaging findings were quantified, and both furosemide doses induced a decrease in mean standard uptake values (SUVmean) compared with the controls (LD 1.46 ± 0.54 and HD 1.05 ± 0.29; controls: 8.90 ± 3.4 [p-value < 0.05]).

**Conclusion:**

We validated a non-invasive, easy, and harmless pre-imaging alternative for decreasing ^18^F-FDG bladder spillover. Our study shows the effect of furosemide on bladder spillover directly on ^18^F-FDG-PET images by measuring SUVmean in the bladder, colon, liver, muscle, and bone marrow.

## Introduction

Clinical and preclinical applications of 2-deoxy-2-[^18^F]fluoro-D-glucose (^18^F-FDG) positron emission tomography (PET) are increasingly used in a wide variety of disciplines such as oncology [1], cardiology [2, 3], neuroscience [4], and inflammatory and infectious diseases [5, 6]. Although unspecific uptake of ^18^F-FDG results in the well-known problem of detectability in several applications [7, 8], accumulation of urine also constitutes a relevant limitation of this radiotracer [9]. The accumulation mechanism depends on the fluorine atom in the molecule, which reduces the affinity of ^18^F-FDG for sodium-glucose linked transporter 1 on the proximal tubules of the nephron, thus decreasing reabsorption [10, 11] and, consequently, increasing elimination of ^18^F-FDG from urine. The accumulation of activity in the bladder leads to spillover, which may hamper visualization of neighboring lesions such as gynecological cancers [12], bladder wall malignances [13], and inflammatory bowel diseases (IBD) [5].

Spillover is more important in small animal research than in human studies owing to the smaller size of the subjects, their faster metabolism, and their higher excretion rates, which lead to rapid bladder refilling [14]. Several methods have been proposed [15] to reduce the ^18^F-FDG signal in the bladder. Non-invasive alternatives are based on the use of saline hydration with or without concurrent administration of diuretic [11, 16]. These methods focus mainly on the evaluation of ^18^F-FDG in urine, but do not explore its actual effects on the PET image, which remain largely unknown. Accordingly, our main goal was to measure the effects of the combination of furosemide and hydration for reducing bladder signal directly on mouse bowel ^18^F-FDG-PET images.

## Materials and methods

All mice were housed in cages under the same conditions (*ad libitum* access to food and water, with 12 h of light and 12 h of darkness).

Nine mice were divided into two groups, the control group (C, n = 4) and treatment group (n = 5). Animals fasted overnight before PET-CT acquisition (ARGUS scanner, SEDECAL, Madrid). Scans were performed under inhaled anesthesia (isoflurane: 5% for induction and 2% for maintenance). Control animals (under no treatment) underwent a single PET-CT study. Two PET-CT images were acquired on different days from the treated mice to evaluate the bladder clearance protocol.

### Bladder clearance protocol

**Fig 1:**
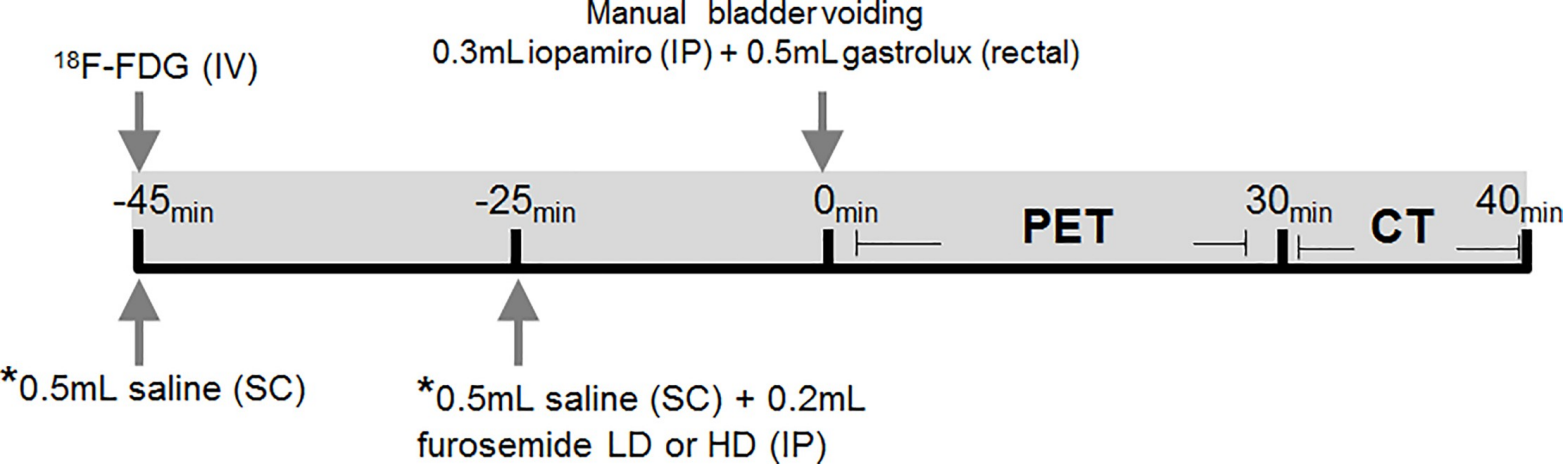
Bladder clearance protocol. Zero time corresponded to the PET acquisition. Forty-five minutes before the scan, the animals received 19.4 MBq of ^18^F-FDG. CT contrasts (iopamiro and gastrolux) were administered immediately before acquisition. Treated animals received two SC saline injections (-45 min and -25 min) and a dose of furosemide (-25 min). *Administration only in treated animals; LD = low dose, 3.5 mg/kg; HD = high dose, 7 mg/kg; SC = subcutaneous; IP = intraperitoneal; and IV = intravenous.

Fig 1 shows the steps followed to prevent accumulation of ^18^F-FDG in the bladder. Treated animals were hydrated by administering two subcutaneous (SC) saline boluses 45 and 25 minutes before image acquisition (time point zero in Fig 1). A single intraperitoneal (IP) dose of furosemide was administered together with the last saline bolus. Two furosemide doses were tested on different days: low dose, LD, 3.5 mg/kg; and high dose, HD, 7 mg/kg; simultaneously with the second saline bolus, 25 minutes before PET acquisition. In both control and treated animals, urination was forced by manual bladder compression to minimize the bladder signal before the animal was placed inside the PET scanner.

### Image acquisition

A 30-minute PET image (prone position) was first acquired followed by a CT image using an X-ray beam current of 240 μA and a tube voltage of 40 kVp. Forty-five minutes before the PET scan, every mouse received an intravenous injection of 19.4 ± 1.8 MBq ^18^F-FDG. Before imaging, the animals also received 0.3 ml iopamidol intraperitoneally (Iopamiro, Bracco Imaging S.p.A, Italy) to delimit the external border of organs and 0.5 ml of gastrolux contrast agent in the rectum [17] (Iberoinvesa Pharma S.L, Spain) to enhance the internal lumen of the colon. PET images were reconstructed using OSEM-2D, with 50 subsets and 2 iterations. CT images were reconstructed using an FDK algorithm [18].

### Image analysis

For each image, regions of interest (ROIs) were drawn on the CT image. These included the bladder, kidneys, liver (0.04±0.01 cc of the left lateral lobe), muscle, small intestine and bone marrow (left ilium). Bladder spillover was quantified as the ratio between SUVmean of distal colon and SUVmean of middle colon. These two different colon areas were selected as follows: a portion of the middle colon as a region at some distance from the bladder and a portion of the distal colon as a region near the bladder. ROIs were transferred to the PET study to measure their mean standard uptake values (SUVmean). Total bladder radioactivity was also measuring to support SUVmean results and evaluate possible bladder volume effects. Additionally, bladder volume (cc) was measured on the CT.

### Statistical analysis

Results are presented as mean ± SD. The Kruskal-Wallis test was used to statistically assess differences in SUVmean between the groups. The Wilcoxon test was used for paired comparisons (LD vs. HD) and Levene test for the variability. Statistical significance threshold was set at p < 0.05.

### Animal ethics statement

Mice were housed in the animal facility of Hospital General Universitario Gregorio Marañón, Madrid, Spain (ES280790000087). All animal procedures conformed to EU Directive 2010/63EU and national regulations (RD 53/2013) and were approved by the local ethics committees and the Animal Protection Board of the Comunidad Autónoma de Madrid (PROEX 204/14).

## Results

**Fig 2:**
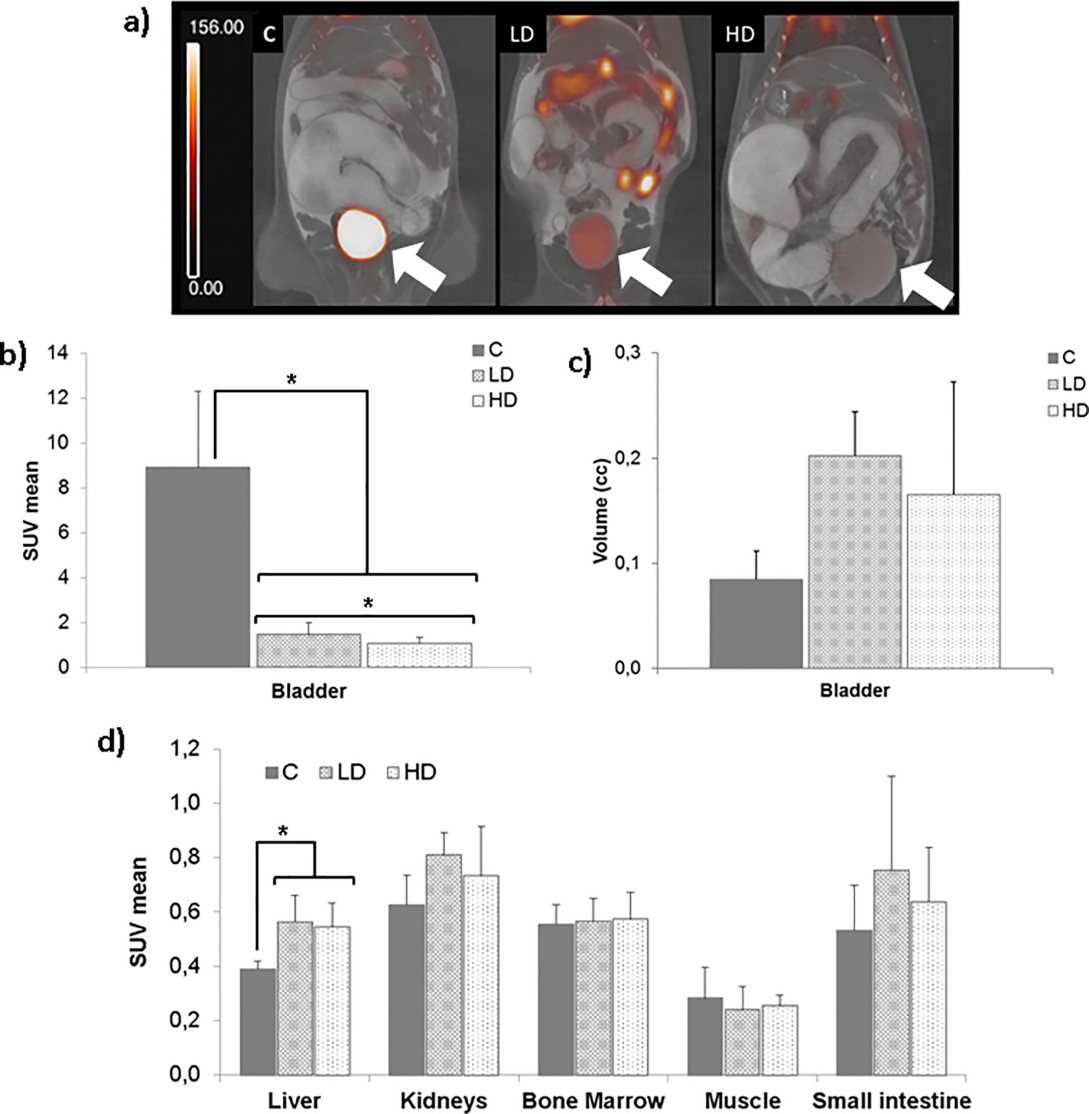
PET-CT images. (A) Coronal PET (red scale, cps x 10^−3^) and CT (gray scale) images of a control animal (C), low dose (LD, 3.5 mg/kg), and high dose (HD, 7 mg/kg). The bladder signal (arrow) is lower in treated animals than in controls. (B) Both furosemide doses led to a reduction in bladder SUVmean compared with the control group. (C) Bladder volume of the treated animals is slightly higher than the controls. (D) Non-significant differences were observed in kidney, bone marrow, small intestine and muscle uptake, although an increase in SUVmean was observed in the liver. *p < 0.05.

Fig 2A shows examples of PET-CT images from each group. ^18^F-FDG-PET activity in the bladder was markedly reduced in both treated groups compared with the control mice. Our visual findings were quantified, and both furosemide doses induced a decrease in bladder SUVmean compared with the controls (LD 1.46 ± 0.54 and HD, 1.05 ± 0.27; controls, 8.90 ± 3.4 [p < 0.05]). Total bladder radioactivity counts also decreased with both furosemide doses (LD 1.49 x 10^5^ ± 4.92 x 10^4^ and HD, 7.40 x 10^4^ ± 3.19 x 10^4^) compared with controls (2.89 x 10^5^ ± 4.28 x 10^4^, [p < 0.05]). In addition, a larger variability was observed in the control group (3.4 vs. 0.54 in LD and 0.29 in HD; [p = 0.012]), coefficient of variation is higher in controls (38.2%) compared with HD (26.0%). The bladder clearance protocol also enabled a clear increase in bladder volume in treated animals compared with the control group, although the difference was not statistically significant (Fig 2C).

No differences were observed for ^18^F-FDG uptake in bone marrow and muscle (Fig 2D). Liver uptake increased in both treated groups (LD, 0.56 ± 0.10; HD, 0.55 ± 0.09) compared with controls (0.39 ± 0.03; p-value < 0.05). A slight increase was also observed in the kidneys and small intestine, although, again, the differences were not statistically significant.

**Fig 3:**
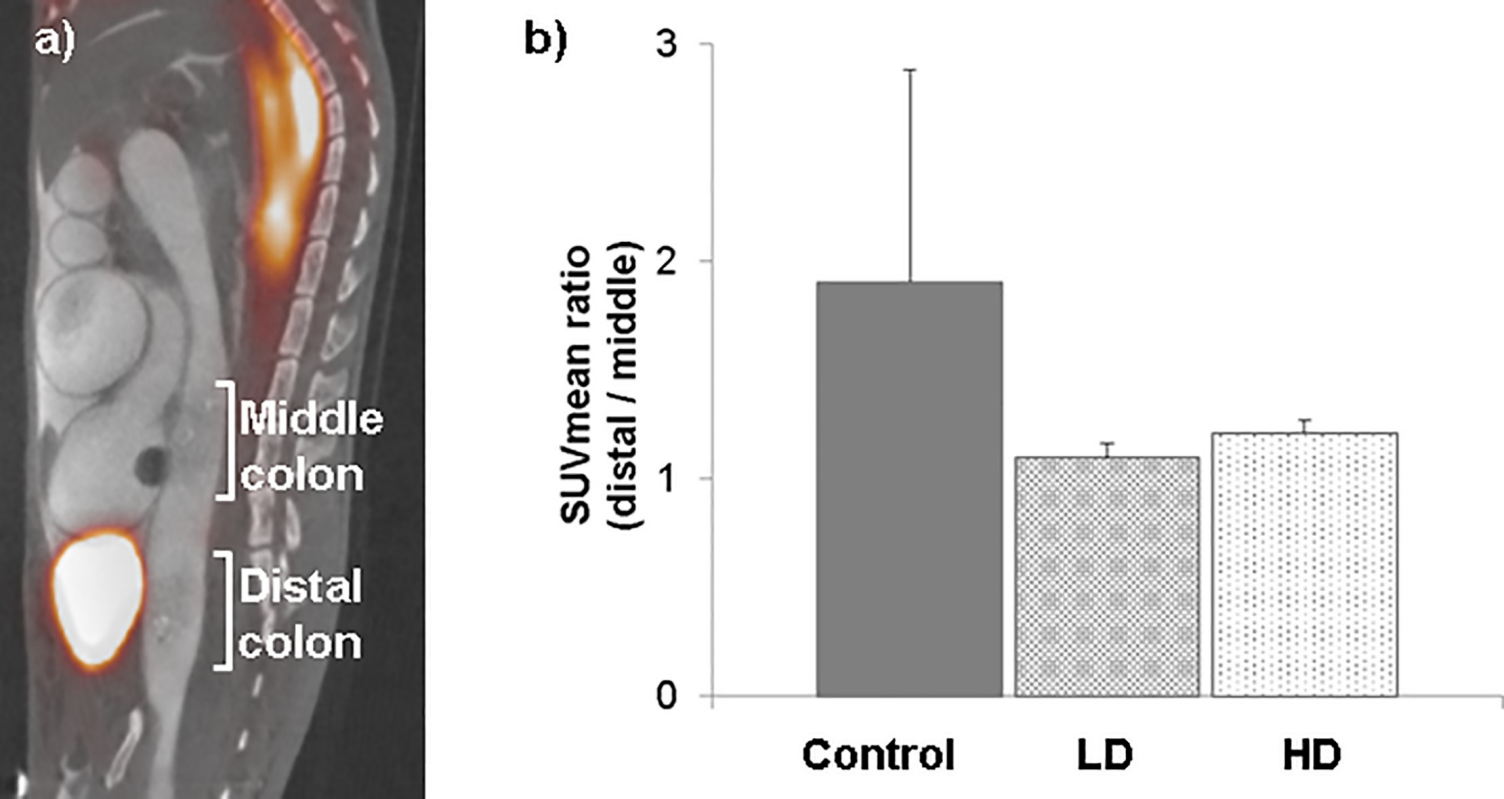
Evaluation of bladder spillover. (A) Sagittal PET (red scale) and CT (grey scale) image of mouse abdomen showing selected areas of the colon. (B) Ratio between the distal colon and middle colon (n = 3 per group). HD, high furosemide dose (7 mg/kg); LD, low furosemide dose (3.5 mg/kg).

Colon uptake ratio (SUVmean of distal colon / SUVmean of middle colon, as explained above) was used to assess the effect of bladder spillover. Fig 3A shows the location of these colon regions in the sagittal plane. Colon uptake ratios were clearly reduced in the treated groups (LD, 1.09 ± 0.06; HD, 1.21 ± 0.06) compared with the control animals (1.9 ± 0.97), thus quantitatively confirming the decrease in bladder spillover when the clearance protocol is applied (Fig 3B). The high dose (7 mg/kg) induced a slight decrease in ^18^F-FDG uptake in the bladder compared with LD (p = 0.043), liver (p = 0.70), kidneys (p = 0.5), and small intestine (p = 0.35). Moreover, bladder volume was smaller than with the low dose (p = 0.5), although the differences were not statistically significant.

## Discussion

In this work we assess a non-invasive, easy, and harmless pre-imaging procedure to decrease accumulation of ^18^F-FDG in the bladder. This clearance protocol combines hyperhydration with a single furosemide dose during the ^18^F-FDG uptake period. Our study quantifies the effect on ^18^F-FDG-PET images by measuring the SUVmean of the bladder bone marrow, liver, muscle, small intestine and colon in order to assess bladder spillover.

Previous studies [11, 16] evaluated the effect of administering a diuretic by directly quantifying ^18^F-FDG in excreted rodent urine under various experimental conditions, such as long pre-hydration periods (IP saline administration 2.5 hours before ^18^F-FDG injection [16] and IV saline infusion before and during the ^18^F-FDG-PET experiment [11]). To our knowledge, our study is the first to quantify the effect of the proposed protocol on ^18^F-FDG-PET images. Our results for bladder uptake also show a decreased standard deviation in treated animals, indicating that the pre-imaging procedure helps to homogenize the studies, as the amount of spillover would be more constant across subjects.

Previous studies in both clinical research [15, 19, 20] and preclinical research [16] concluded that the key to obtaining satisfactory bladder clearance is the optimal time of furosemide injection with respect to ^18^F-FDG administration [15, 16, 19, 20], although this timing has not yet been established in rodent ^18^F-FDG-PET imaging. We had observed that the bladder clearance protocol (saline with furosemide) takes effect 15–20 minutes after administration (S1 Fig). Additionally, in rodents, excretion of ^18^F-FDG occurs during the first 30 minutes after administration, with decreased blood levels [11]. Taking both observations together (optimal time for furosemide and ^18^F-FDG excretion) we chose to administer furosemide 25 minutes after the ^18^F-FDG injection, assuming that the diuretic effect of furosemide begins close to the moment when the ^18^F-FDG blood concentration decreases. Our hypothesis was confirmed after analyzing the ^18^F-FDG-PET images, because the timing selected reduced the bladder signal (Fig 2A), with no marked effects on surrounding tissues such as muscle, small intestine and bone marrow (Fig 2D).A secondary effect of furosemide is the increased bladder volume, well described in patients and that may be helpful for the visualization of bladder wall malignances [15]. However, in our study we observed that bladder volume increase may compress and displace adjacent organs, such as the colon, thus hampering visualization. Despite this possible negative effect, we are already routinely using the high furosemide dose combined with hyperhydration in mouse models with abdominal PET imaging, such as Crohn’s disease, infectious colitis and prostate cancer.

The excellent bladder suppression we achieved is consistent with the observations of Kosuda et al. [16], who reported an increase in ^18^F-FDG uptake in several tissues (liver, kidney, small intestine, heart, etc.), which agree with our findings (Fig 2D). Kidney and liver are the organs that are most susceptible to furosemide [21]. Foy et al. [22] showed a significant reduction in liver glycogen 1 hour after IP administration of the 200 mg/kg dose of furosemide, possibly indicating an increase in liver metabolism and, therefore, an increase in ^18^F-FDG uptake. We did not observe any difference between the groups for uptake of ^18^F-FDG in bone marrow, small intestine and muscle.

To evaluate the effects of bladder clearance, we measured uptake of ^18^F-FDG in the distal and middle colon (Fig 3). Both areas have equivalent ^18^F-FDG uptake (ratio close to one), whereas the control animals showed almost double the uptake ratio in the distal colon. Therefore, the protocol we propose clearly reduces bladder spillover in the colon.

Interpretation of the data in this study is limited by the fact that mice are the rodent species that is most sensitive to furosemide-induced toxicity [21]. Our protocol increases bladder volume, which may compress and displace adjacent organs, such as the colon, thus hampering visualization. We observed this problem in four treated animals, forcing us to reduce the sample size per group for the spillover analyses. This undesirable effect may be reduced by forcing urination before placing the animal inside the PET-CT device although in our case was not effective in all the animals. Diuretic drugs reduce blood pressure, and this must be taken into account if the animals are under treatment with additional drugs. Finally, it must be noted that the decrease in bladder activity may contribute to an overall better contrast recovery by increasing other organs uptake, although this effect depends on the system noise-equivalent counting rate (NEC) and is expected to be small for most commercial PET scanners at the dose levels used here.

In conclusion, we characterized a non-invasive and effective pre-imaging protocol that reduces accumulation of ^18^F-FDG in urine. Our pre-imaging protocol is based on the combination of hyperhydration and furosemide during the ^18^F-FDG uptake period. With the exception of the liver, our pre-imaging protocol did not reveal alterations in tissue uptake measured directly on the PET images. Furthermore, we demonstrated that it is an effective method for decreasing bladder spillover in neighboring organs such as the colon.

## Supporting information

S1 Fig Urination effects of hydration and furosemide. (a) Minute 0 was chosen as reference coinciding with the furosemide administration in treated groups. Every animal received two subcutaneous (SC) saline boluses at minutes -20, and 0. *Treated groups (LD and HD) additionally received an intraperitoneal (IP) bolus of furosemide (0-time, total volume of 0.2 ml). (b) Time course of voiding. Points represent means of three mice per group. LD: low dose of furosemide group (3.5 mg/kg) and HD: High dose of furosemide group (7 mg/kg).
(TIF)
