# Supplementary figures and images for: Suppression of 18F-FDG signal in the bladder on small animal PET-CT
Source: PLoS One. 2018 Oct 17;13(10):e0205610. doi: 10.1371/journal.pone.0205610 (PMC6192599; doi:10.1371/journal.pone.0205610)

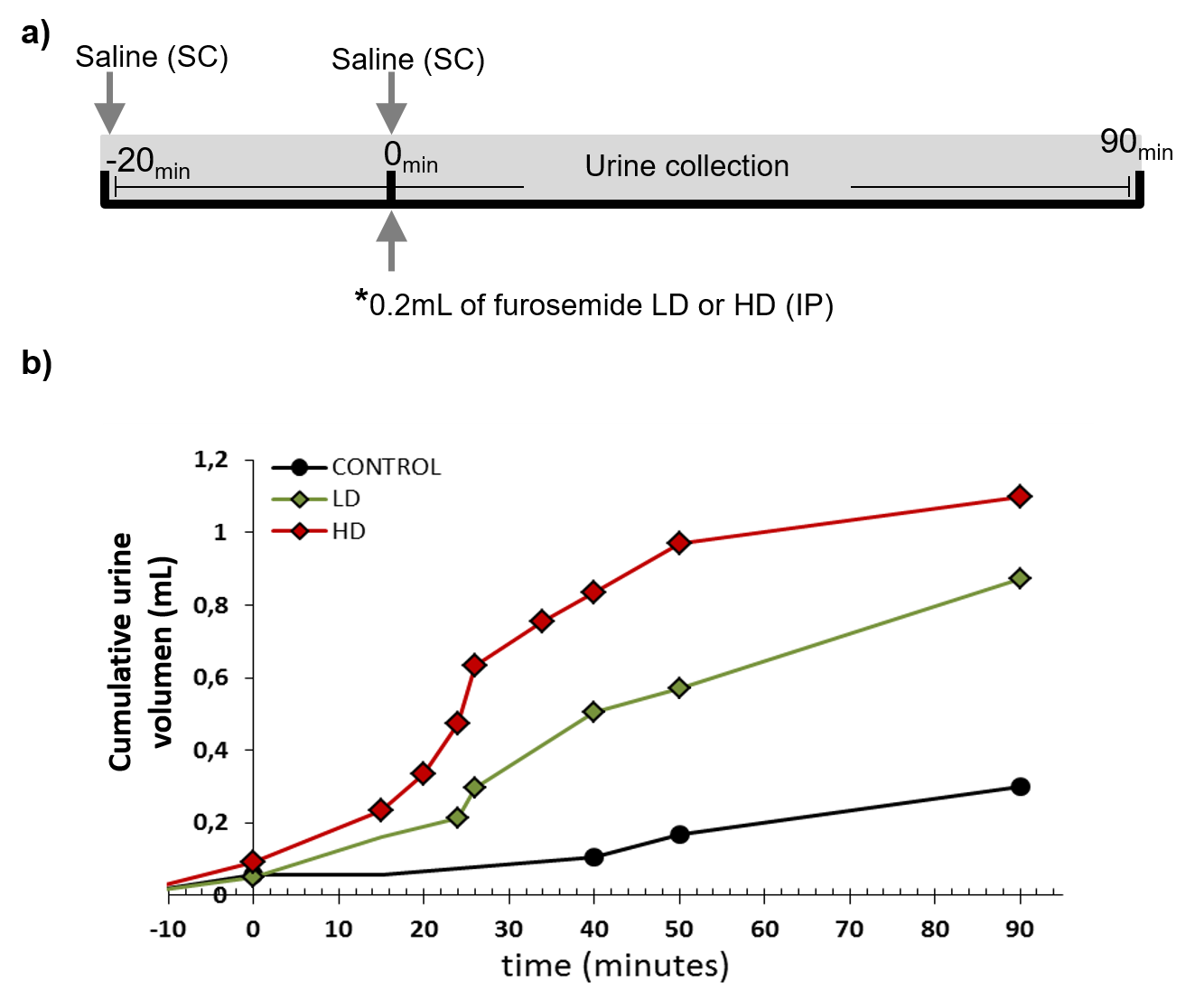

Supplement: S1 Fig — (a) Minute 0 was chosen as reference coinciding with the furosemide administration in treated groups. Every animal received two subcutaneous (SC) saline boluses at minutes -20, and 0. *Treated groups (LD and HD) additionally received an intraperitoneal (IP) bolus of furosemide (0-time, total volume of 0.2 ml). (b) Time course of voiding. Points represent means of three mice per group. LD: low dose of furosemide group (3.5 mg/kg) and HD: High dose of furosemide group (7 mg/kg). (TIF) [file pone.0205610.s001.tif]
